# Chats about Medical Museums

**Published:** 1887-03-26

**Authors:** 


### CHATS ABOUT MEDICAL MUSEUMS.

By One of the Crowd.

II.—St. Thomas's Hospital.

There is no medical museum in London at all comparable in extent or interest to that which has been the subject of a previous notice—the Museum of the Royal College of Surgeons in Lincoln's Inn Fields. But for the existence of this great central institution, such a museum as that attached to St. Thomas's Hospital would be a notable one. The museum building is a large and lofty hall, with two galleries, one above the other, running all round it. These are reached by external staircases, so that the entire interior space of the building is available for the display of a marvellous collection of objects, few of which are suitable for description in an article for miscellaneous readers, as, for the most part, they illustrate diseases, all of which of course are painful to contemplate, and many of which are very hideous, as may be expected in a collection of this nature.

In a medical museum, it need hardly be said, there are some skeletons. Compared with the grinning company in Lincoln's Inn Fields, however, they are few. The most noteworthy of them is that of a South Australian, with a formidable-looking shillelah in the case beside him. He appears to have been a person of some repute in his day, having been the death of a good many people with this cudgel of his. Some readers may perhaps recall to mind the doughty deeds of Nulla Nulla. Here he is, doing penance in a glass case. He got quietly away in his grave once, but luck was against him, and ferreted him out again, to have his bones bared and his joints wired, and thus to be converted into an object of permanent interest, and some little commercial value.

By the way, it is an odd reflection that many a poor wretch now limping about the world literally "not worth a penny," would, if he could only adopt Sydney Smith's suggestion for a very hot day—take off his flesh and go in his bones—immediately have an actual market value of from five to ten or twelve pounds. A skeleton of a sound constitution and a good colour is always worth a ten-pound note.

The human framework is a fearful and wonderful thing in some of its possible forms and phases. There are human bones here from which all the animal matter has dwindled away—atrophy of the bone, the malady is designated—and which are mere shells, as thin almost as egg-shells. There are others which, from some mysterious cause, have thrust out growths—bones growing out of bones, in the most capricious and extraordinary way. The mortal relics of one poor fellow afflicted in this way are shown in a glass case. Suicide terminated his sufferings. He threw himself in front of a railway train, and was caught by the buffer of the engine and killed, but, marvellous to relate, not a bone of his body was broken. This disorder—exostosis, a sort of bony tumour—is not an unfamiliar phenomenon, but such manifestations of it as are shown in this case must, happily, be very rare. Many of the bones exhibited here are in a condition which renders it easy to understand how apt some people's bones are to break upon the smallest occasion. Perhaps the most remarkable instance of this aptitude ever met with was presented in the framework of a man whose skeleton was a few years ago sent up from some county hospital—the Sussex County Hospital, if we remember aright—to the collection got together at Burlington House on the occasion of the International Medical Congress held in London. This unlucky individual was born with both arms broken, and all his life long he seemed to be every now and again breaking himself in some fresh place. His bones seemed to be as brittle as glass. Notwithstanding his curious infirmity, he managed to live to an age of somewhere about sixty. He got married at thirty years of age, and he is said to have stood then at a height of five feet four inches. But ten years later what little strength he had seems to have collapsed, and though he lived on, the constant breaking and contortions of his bones reduced him to 39½ inches, and his skeleton stood something less than this. He had two children; and they are both said to have inherited the paternal weakness.

To return to St. Thomas's Museum. There is one series of objects here which bring the visitor back to the grimmest realities of the most frighful of the world's military struggles. In a glass case are a number of bullets, fragments of exploded shells, and other objects of the kind, brought from the battle-fields of the Franco-German war—many of them having been embedded in the limbs and bodies of the wounded or the slain in that frightful contest. In a contiguous glass case are some of the fractured bones brought from the battle-field, or subsequently taken from the bodies of soldiers who had been wounded there. There are one or two frightful smashes, and in one of the broken limbs may still be seen the bullet which did the mischief, embedded right in the bone. Notwithstanding the frightfully shattered condition of the limbs, it is evident the owners of them must have lived for a considerable period after receiving the injury, from the fact of large growths of new bone being presented. These objects seem to bring one more immediately into those scenes of terrible slaughter than the most realistic of pictures could do.

The most conspicuous objects in this museum are two life-sized models, one of wax and the other of plaster-of-paris, representing the bodies of two emaciated mortals who have died from diseases which have distorted their limbs and wasted their flesh in the most remarkable manner. The wax model is a particularly realistic representation of a woman who has been afflicted with what is known as Charcot's disease, and whose knee-joints are very curiously twisted. These are on the ground-floor. In one of the galleries are some very remarkable "preparations" of children malformed at birth and before it. Perhaps it is not desirable to refer to these more minutely; but there is one object which probably is absolutely unique, and which cannot very well be entirely ignored in a notice of the contents of this museum. This is the body of a child which the mother for some thirty or forty years always believed herself to be pregnant with, but in the existence of which nobody else believed till after her death, when examination proved she was correct. It is believed that no other such case has been met with in the whole history of obstetrical science.

